# Comparison and application of depression screening tools for adolescents: scale selection and clinical practice

**DOI:** 10.1186/s13034-025-00908-2

**Published:** 2025-05-09

**Authors:** Xinyu Li, Wei Jin, Lu Han, Xingyu Chen, Lihong Li

**Affiliations:** 1 The Second School of Clinical Medicine, Zhejiang Chinese Medical University, Hangzhou, 310053 China; 2 Department of Acupuncture, The Second Affiliated Hospital of Zhejiang Chinese Medical University, Hangzhou, 310005 China; 3 Jinhua Academy of Zhejiang Chinese Medical University, Jinhua, 310053 China

**Keywords:** Adolescents, Depression, Screening scales, Diagnostic tools

## Abstract

**Background:**

Clinical assessments rely primarily on patients’ emotional expressions and scale scores. However, due to cognitive differences and the complexity of emotional expression among adolescents, existing assessment tools often present challenges in their selection and application. This study reviews and analyzes the literature related to 8 commonly used adolescent depression assessment scales, including the Hamilton Depression Scale (HAMD), the Beck Depression Inventory (BDI), the Center for Epidemiologic Studies Depression Scale (CES-D), the Reynolds Adolescent Depression Scales (RADS), the Children’s Depression Inventory (CDI), the Kutcher Adolescent Depression Scale (KADS), the Patient Health Questionnaire (PHQ) and the Depression Screener for Teenagers (DesTeen). Through a comprehensive analysis of each scale’s strengths, limitations and practical applications, this narrative review aims to guide healthcare practitioners and researchers in selecting optimal measurement tools for different clinical and research contexts.

**Methods:**

Relevant studies on 8 frequently used or well-supported adolescent depression assessment scales (CDI, RADS, CES-D, BDI, PHQ, KADS, HAMD, DesTeen) were retrieved from PubMed, Web of Science, CNKI, and Wanfang databases. A total of 102 articles were ultimately selected for data extraction to determine the reliability and validity of these scales. Additionally, 13 original development studies of the included scales were further reviewed to extract and analyze information on their developmental background, structural dimensions, item composition, and applicability.

**Results:**

Recent studies on depression assessment scales have focused on the development of precise diagnosis and personalized evaluation. All 8 adolescent depression assessment scales generally exhibit good reliability and validity. Among them, the HAMD is used for detailed clinical evaluation of depressive symptoms but suffers from complexity due to its reliance on professional assessors. The BDI and the CES-D provide the most comprehensive dimensions. While the BDI is suitable for clinical assessments, it has the drawback of containing items that may be difficult to understand. The CES-D is well-suited for epidemiological research and large-scale screenings but has the limitation of unclear differentiation between emotional and somatic symptoms. The RADS is recognized for its comprehensive items and high reliability and validity, although its lengthy items may lead to respondent fatigue. The CDI allows multidimensional assessment of depressive symptoms but has been debated regarding its applicability across different age groups. The KADS, explicitly designed for adolescents, is a promising tool; however, its relatively recent development has resulted in limited validation studies. The PHQ is appropriate for rapid screening and tracking treatment effects but lacks sufficient emotional evaluation. The DesTeeen, designed for adolescents, features concise and clear item phrasing, but it’s only available in the German language.

**Conclusions:**

The 8 standard scales demonstrate high accuracy in screening adolescent depression, but challenges persist in selecting scales for different contexts and ensuring their cross-cultural validity.

## Introduction

 Adolescent depression represents a critical global public health challenge, significantly impacting the youth mental health, physical wellbeing, and social functioning [1]. With a prevalence rate of 6.2% [2], it is the leading cause of illness and disability among adolescents [3]. This condition imposes substantial socioeconomic and clinical burdens, manifesting in cognitive dysfunction, academic decline, substance use disorders, and suicidal behaviors [4, 5]. Alarmingly, suicide has emerged as a primary cause of mortality among adolescents [3]. Given that the pathophysiology of adolescent depression is not yet fully understood and there is a lack of diagnostic biomarkers, clinical assessments rely primarily on patients’ emotional expressions and scale scores [1]. However, the unique developmental stage of adolescents results in differences in scale understanding, and clinical symptom presentations differ from those in adults [6], which impacts the accuracy of depression assessment scales [7]. Consequently, existing methods for diagnosing and developing treatment plans for adolescent depression have limitations regarding applicability, diagnostic bias, and intervention guidance. Therefore, selecting the appropriate scale is a crucial issue that needs to be addressed in clinical settings.

Multiple assessment tools are currently available for evaluating adolescent depression, including the Hamilton Depression Scale (HAMD), the Beck Depression Inventory (BDI), the Center for Epidemiologic Studies Depression Scale (CES-D), the Reynolds Adolescent Depression Scale (RADS), the Children’s Depression Inventory (CDI), the Kutcher Adolescent Depression Scale (KADS), the Patient Health Questionnaire (PHQ) and the Depression Screener for Teenagers (DesTeen). However, these instruments vary significantly in their dimensional structure, administration duration, and target age ranges [8], selecting the most suitable assessment tool for specific clinical or research applications become a major challenge in practical work. To address this issue, this narrative review compares and analyzes 8 widely used adolescent depression screening instruments based on 102 published articles up to October 10, 2024. Our analysis examines their item composition, implementation characteristics, and practical applications. The findings aim to provide evidence-based recommendations for healthcare professionals and researchers in selecting appropriate screening tools for adolescent depression assessment.

## Methods

### Literature search

This narrative review is based on a search of Chinese and English literature databases regarding adolescent depression scales. A comprehensive search was conducted in PubMed and Web of Science using search terms such as “depression“[Mesh] AND “adolescent“[Mesh] AND (instrument* OR measure* OR scale* OR evaluate*), and in CNKI and Wanfang databases with keywords such as “青少年(adolescent)” AND “抑郁症(depression)” AND “量表(scale)”. Literature searches were conducted through October 10, 2024. The narrative review process involved initial screening of titles and abstracts, followed by comprehensive full-text evaluations. Reference lists of relevant publications were manually examined to ensure comprehensive literature coverage.

### Scale identification and original literature tracing

During the initial and secondary screening of both Chinese and English literature, the following criteria were used to identify adolescent depression scales: (1) the scale must assess depression (excluding scales for specific types of depression such as post-stroke depression or postpartum depression); (2) the scale must apply to adolescents. Scales that did not meet these criteria were excluded. After identifying the relevant scales, further searches were conducted using each scale’s full name and abbreviation to trace the original literature that developed the scale.

### Study selection

To guarantee the quality and representativeness of the included studies, a rigorous selection process was implemented. Two independent researchers conducted separate literature searches and subsequently compared and discussed the results. It was explicitly stated that studies with negative findings or conflicting results would be included. Studies with significantly missing data, which could not be supplemented by contacting the authors, was considered for exclusion. In cases where the two researchers had differing opinions, a third researcher reviewed the results to make the final decision. This multi-tiered approach minimized bias and ensured that the most relevant literature was incorporated.

### Information extraction and review

The original development literature of the identified scales was reviewed and analyzed to extract key information such as the year of development, complete and abbreviated names of the scale, primary developers, and the country of origin. The dimensions of each scale were orderly organized and summarized to ensure their applicability in a multidimensional assessment of depressive symptoms. Additionally, the construction of the depression descriptions in each scale, including the dimensionality, item settings, scoring methods, and limitations, was deeply analyzed and summarized.

## Analysis of the 8 common screening scales

### Hamilton depression rating scale (HAMD)

The HAMD was first proposed by British psychiatrist Max Hamilton in 1960 and is regarded as the “gold standard” for evaluating the severity of depression in clinical trials of antidepressant treatments [9]. Items are rated on a scale of 0–2 or 0–4 to reflect symptoms severity, with higher scores indicating greater depressive severity. Predominantly items are scored on a 0–4 scale. The assessment method combines interview and observation, typically conducted by two interviewers: one leading the interview and the other posing follow-up questions at the end. The raters independently assign scores, and these are aggregated to provide a quantitative measure of depressive severity [10].

The HAMD is available in multiple versions. The original 21-item version was later refined by Hamilton, who noted that the last four items—related to diurnal mood variation, depersonalization, paranoid symptoms, and obsessive-compulsive symptoms—did not accurately capture the core symptoms of depression and should be excluded from the total score [9]. Consequently, a 17-item version was introduced in 1976, becoming the predominant variant [11, 12]. The HAMD is also expanded to a 24-item version (HAMD-24) with the addition of three items assessing helplessness, hopelessness, and worthlessness, enhancing its symptom coverage [11]. Although the sources of these extra items are obscure, the 24-item version was referenced in an 1981 study comparing cognitive and pharmacological therapies [13].

The HAMD is extensively applied in clinical practice with adolescents [14–17], but there is a paucity of studies on its reliability and validity in this demographic. The HAMD has well - established reliability and validity data in adults and suggest potential applicability to adolescents. Howeverthe HAMD-17 demonstrates robust internal reliability, inter-rater reliability, and test-retest reliability in adults, but exhibits weaker reliability at the item level [18], and has faced criticism for conceptual and psychometric limitations [18, 19]. For example, its unidimensional structure may not sensitively reflect nuanced symptom changes, such as guilt or suicidal ideation [11, 20].These limitations, observed in adults, could be further amplified in adolescents due to developmental differences in symptom expression and cognitive processing, underscoring the need for age-specific validation studies.

To provide a more comprehensive assessment of depressive symptoms, the HAMD-24 includes additional items, but introduces the complexity and difficulty of administration, particularly concerning scoring consistency and accuracy [21]. Based on adult reliability and validity studies, it is hypothesized that the HAMD may also exhibit certain reliability and validity in adolescents, but further validation is essential. Currently, cross-cultural validation of the HAMD in adolescents is sparse, with adult studies being more prevalent. The HAMD has been validated in multiple languages, including French [22], Turkish [23], Arabic [24], and Chinese [25], primarily in adult populations. Given the strong reliability and validity of the HAMD across various languages and cultural contexts, it is reasonable to infer that cross-cultural validation in adolescent populations is feasible. This provides a theoretical basis for the future application of the HAMD in adolescent depression assessment and supports its potential use in this demographic.

### Beck’s depression inventory (BDI)

The BDI was initially formulated by American scholar Aaron T. Beck in 1961 for the assessment of depressive symptoms in adults [26]. The original inventory is presented in two versions: a 21-items and a 13-items, with respondents asked to self-assess based on their experiences within the preceding week. A elevated total score indicates a greater severity of depression [27]. The structural model of the BDI has been shown to exhibit either a two-factor or three-factor structure, depending on the study population [28]. A principal component analysis extracted four main factors: depressive mood, low self-esteem, bodily disturbances, and social withdrawal. These factors encapsulate distinct dimensions of depression [29].

In adolescent populations, the validity of the BDI has been confirmed through various studies. One study involving 122 adolescent patients with depression reported an internal consistency of 0.91. When BDI scores exceeded 13, the sensitivity, specificity, and positive predictive value (PPV) for diagnosing major depressive disorder (MDD) were 86%, 82%, and 83%, respectively. These metrics improved to 89%, 88%, and 93%, respectively, after a two-week interval [30]. These findings demonstrate that the BDI is highly reliable for monitoring the severity of depressive symptoms and treatment outcomes in adolescents.

The Beck Depression Inventory, Second Edition (BDI-II), was revised in 1996 to correspond with the diagnostic criteria for depression in the fourth edition of the Diagnostic and Statistical Manual of Mental Disorders (DSM-IV) [31]. As one of the most widely used self-report depression scales, the BDI-II not only updates its items content to align with DSM-IV criteria but also extends the evaluation period to two weeks to better reflect the severity of persistent depressive symptoms. The BDI-II consists of 21 items, each scored on a 0 to 3 scale, for a total maximum score of 63. Based on the total score, depression severity is categorized as follows: no depression (0–13), mild depression (14–19), moderate depression (20–28), and severe depression (29–63).

Several studies have validated the psychometric properties of the BDI-II. A systematic review of 118 relevant studies revealed that the average internal consistency estimate for the BDI-II was 0.90, with test-retest reliability ranging from 0.73 to 0.96, all higher than the original BDI [32]. Among 1,072 Korean adolescents, the internal consistency estimate for the BDI-II was 0.89. Furthermore, its correlation coefficient with the PHQ-9, a widely recognized screening tool for adolescent depression, was 0.75, suggesting strong concurrent validity of the BDI-II when compared to an established instrument [33].

The Chinese version of the BDI-II (BDI-II-C) was translated from English to better align with Chinese linguistic expressions and cultural context. A study with 6,057 high school students in Changsha assessed the reliability and validity of the BDI-II-C. It showed that the internal consistency (Cronbach’s α) for non-clinical and depressed adolescents was 0.89 and 0.93, respectively, with test-retest reliability of 0.76 at one week and 0.56 at two months [34]. The BDI-II-C was able to distinguish among varying degrees of depression severity. Still, the false positive rate for adolescent depression screening was 17%, suggesting that the BDI-II-C has a relatively high false positive rate for depression screening[35]. It is deemed appropriate for monitoring depressive symptoms but not for clinical diagnosis.

### Center for epidemiologic studies depression scale (CES-D)

The CES-D was developed by Radloff in 1977 under the auspices of the U.S. National Institute of Mental Health. Its development drew upon existing tools, including the BDI, the Self-Rating Depression Scale (SDS), and the depression subscale of the Minnesota Multiphasic Personality Inventory (MMPI). The CES-D assesses the frequency of depressive symptoms or feelings over the past week through 20 items organized into four factors: depressed affect, positive affect, somatic complaints, and interpersonal difficulties [36]. Each item is scored on a 4-point Likert scale (0–3), with four items requiring reverse scoring. The total score ranges from 0 to 60, with a clinical cutoff point of 16 commonly employed for screening [37]. The scale requires a reading level equivalent to the sixth grade [38].

A distinctive feature of the CES-D is its suitability for epidemiological studies, focusing primarily on the frequency of depressive mood or affect rather than the entirety of the depressive syndrome. This design makes it particularly effective for cross-sectional comparisons at different time points.

The CES-D has been extensively validated for adolescents and demonstrates excellent internal consistency, with Cronbach’s α coefficients ranging from 0.88 to 0.89 [34, 38, 39]. A study involving 16,047 Chinese adolescents aged 11 to 18 reported a Cronbach’s α of 0.88 and a test-retest reliability coefficient of 0.49 over an eight-week interval [39]. Moreover, research conducted by Yang Wenhui compared the validity and cutoff scores of the CES-D and the BDI-II-C for screening depression in Chinese adolescents [40]. This study found that the CES-D exhibited robust criterion validity, with higher validity at optimal cutoff scores than traditional ones. However, the CES-D demonstrated a relatively high false-positive rate (21–26%), indicating its potential to generate false positives during screening. Consequently, it is more suitable for identifying depressive symptoms rather than diagnosing clinical depression.

The CES-D has a derivative version, the CES-DC (Center for Epidemiologic Studies Depression Scale for Children), developed by Weissman et al. in 1980 to accommodate children’s and adolescents’ reading and comprehension levels. By utilizing more straightforward language, the CES-DC aims to assess depressive symptoms in younger populations more accurately [41]. In the United States and Europe, the CES-DC is widely used for screening depression among children and adolescents and has demonstrated good reliability and validity in various epidemiological studies [42]. However, its cross-cultural applicability requires further investigation.

A recent study evaluated a Rwandan version of the CES-DC in a sample of 367 adolescents aged 10 to 17 from Kayonza District, southeastern Rwanda. The scale exhibited high internal consistency (Cronbach’s α = 0.86) and test-retest reliability (0.85). Using MINI KID(Mini International Neuropsychiatric Interview for Children and Adolescents) MDE(Major Depressive Episode) diagnosis as the gold standard, the CES-DC achieved an area under the ROC curve of 0.825, with sensitivity and specificity values of 81.9% and 71.9%, respectively [42].

### Reynolds adolescent depression scales (RADS)

The RADS was developed by American psychologist William M. Reynolds in 1981. It was designed as a self-report measure for adolescents aged 12–18, based on the DSM-III, Research Diagnostic Criteria (RDC), Weinberg Criteria, and the Hamilton Depression Rating Scale [43]. The RADS evaluates depressive symptoms across four domains: dysphoric mood, anhedonia/negative affect, negative self-evaluation, and somatic complaints. It consists of 30 items scored on a 4-point frequency scale: 1 (Never), 2 (Sometimes), 3 (Often), and 4 (Always). Total scores range from 30 to 120, with a clinical cutoff score of 77. Following several revisions, a normative sample was established in 1985 based on data from over 12,000 high school students across the United States [44]. The item content is closely aligned with adolescents’ daily lives and experiences, making the RADS particularly suitable for use in school-based populations.

Reynolds and his colleagues conducted multiple validation studies with sample sizes ranging from 126 to 2240 participants, including both non-depressed and clinically depressed adolescents. Results indicated excellent internal consistency, with Cronbach’s α coefficients ranging from 0.92 to 0.96 [43, 45, 46], underscoring the RADS’s high reliability in assessing depressive symptoms among adolescents. The RADS provides a comprehensive reflection of the physical and emotional experiences of depression, with a breadth of content that captures various dimensions of depressive experiences more thoroughly than many other scales. Due to its relatable item content, the RADS is particularly well-suited for screening depression in school classrooms. It can also be used clinically to assess the severity of depressive symptoms and monitor treatment outcomes. Furthermore, the RADS has been widely utilized in theoretical research on the structure of depression [44].

The second edition of the Reynolds Adolescent Depression Scale (RADS-2) was published in 2002, incorporating data from a new standardized sample reflecting the 2000 U.S. Census. RADS-2 retained the original 30 items but expanded the age range to 11–20 years and required a minimum reading level equivalent to the third grade. The scale includes four subscales derived from its factors, comprehensively assessing depressive symptoms across various domains. The Cronbach’s α for the standardization sample was 0.93, with subscale internal consistency ranging from 0.80 to 0.87. In a two-week test-retest study involving 1765 adolescents, the RADS-2 total depression score demonstrated a reliability coefficient of 0.85, while subscale coefficients ranged from 0.77 to 0.84 [47].

Additionally, the RADS-2 exhibits significant correlations with clinical interviews and other self-reported depression measures, such as the HAMD. The RADS-2 has also shown strong applicability in cross-cultural contexts. For example, a study assessing the Korean version of the RADS-2 with 1,765 Korean adolescents reported a Cronbach’s α of 0.93 and a test-retest reliability of 0.85, demonstrating high reliability and stability. The scale effectively assessed depressive symptoms in Korean adolescents, with results supporting its cross-cultural validity and suitability for this population [48].

### Children’s depression inventory (CDI)

The CDI, developed by American psychologist Kovacs in 1977 and modeled after the Beck Depression Inventory, is a self-report scale for assessing depressive symptoms in children and adolescents [49]. It comprises five subscales: low self-esteem, negative mood, lack of pleasure, inefficacy, and interpersonal difficulties. Each item has three different levels of depression severity (occasional, often, always), with a score range of 0 to 2 and a maximum total score of 54. A cutoff of 19 is used to identify depressive symptoms. Additionally, parent and teacher versions are available to enhance the accuracy of identifying children with depressive symptoms [5]. The CDI’s advantages include straightforward language, short completion time, and robust psychometric properties. However, its specificity is relatively low, and it performs moderately when distinguishing depression from other affective disorders, such as anxiety [50].

A systematic review encompassing 14 studies involving 7372 participants reported an average internal consistency reliability coefficient of 0.86. Sensitivity and specificity estimates were 0.83 and 0.84, respectively, revealing high heterogeneity across studies. Prudence is warranted when using the CDI across various clinical settings, as the positive predictive value (PPV) in clinical samples ranged from 0.21 to 0.90. This suggests the potential for a certain proportion of false positives when predicting depressive symptoms [8]. Cultural adaptability of the CDI is a significant concern. For example, a study employing Multi-Group Confirmatory Factor Analysis (MGCFA) validated the CDI in second and third-grade students from China and Italy, finding significant differences in factor variances and covariances between the two countries’ samples [51]. This indicates the necessity of carefully considering cultural differences when applying the CDI to assess depressive symptoms in adolescents across different cultural contexts.

Several versions of the CDI have been developed to meet varying contextual needs. The shortened version, CDI-S (Children’s Depression Inventory-Short Version), is a unidimensional, quick assessment tool consisting of 10 items. Exploratory Factor Analysis (EFA) conducted on 809 children aged 8 to 12 years revealed that the variance in CDI-S scores is predominantly explained by a general factor, rendering it appropriate for use as a singular measure. Convergent validity studies show that CDI-S correlates highly with another depression measure, the RCADS-MDD, demonstrating good convergent validity. Additionally, its moderate correlation with the Spence Children’s Anxiety Scale (SCAS) suggests its effectiveness in distinguishing between depression and anxiety symptoms [50].

The revised version of the CDI, known as CDI-2, was crafted to accommodate fluctuations in depression levels over time and to address the requirement for a re-standardized questionnaire. It consists of 28 items. Studies have shown that its Cronbach’s α coefficient is 0.91, and its short-term (2–4 weeks) test-retest reliability coefficient is 0.89. Statistical tests for structural validity also demonstrate excellent fit indices [52].

### Kutcher adolescent depression scale (KADS)

The KADS was developed by Professor Stanley Kutcher at Dalhousie University, Canada, as a self-report measure for screening depressive symptoms in adolescents. It has three versions: KADS-16, KADS-6, and KADS-11. The original KADS-16 consists of 16 items that correspond to the core depressive symptoms in adolescents, designed better to assess the frequency or severity of these symptoms. Fourteen of these items use a 0–3 scale to indicate the frequency of specific symptoms in the past week: “not at all,” “often,” “most of the time,” and “all of the time.” One item concerning “suicidal or self-harming thoughts or behaviors” is rated on a 0–4 scale. The KADS-6 and KADS-11 are derived from the KADS-16, containing 6 and 11 items, respectively [53]. All items assess specific depressive symptoms, such as anhedonia and emotional symptoms.

A distinctive feature of KADS is its emphasis on core depressive symptoms. It is characterized by good predictive or criterion validity and strong psychometric properties. Its straightforward language and ease of use make it suitable for epidemiological studies and clinical research [54].

Although the overall assessment of KADS remains relatively limited, a study applying the KADS for screening adolescent depression in schools found that the KADS-6 outperformed both the BDI and KADS-16. With a cutoff score of 6, the KADS-6 demonstrated high sensitivity (92%) and specificity (71%) for screening purposes [54]. Another study, which used KADS-11 to screen 3180 middle and high school students aged 11 to 17 from various provinces in China, reported an internal consistency (Cronbach’s α) of 0.84. The correlation between individual items and the total score was statistically significant at the 0.01 level, and the split-half reliability and one-month test-retest reliability were both 0.77. These findings suggest that KADS has good applicability for assessing depressive symptoms in adolescents [55].

However, due to cultural and linguistic differences, research on the cross-cultural applicability of KADS still needs to be improved, and further studies and validation are required.

### Patient health questionnaire (PHQ)

The PHQ is a simplified self-administered version of the PRIME-MD, addressing the limitations of the latter, such as its lengthy administration time and reliance on clinicians. By allowing patients to complete it independently, the PHQ offers high efficiency and practicality [56, 57]. Since its introduction, the PHQ has developed into several versions, including the PHQ-2, PHQ-8, and PHQ-15, to meet the screening needs of different contexts. Among these, the PHQ-9 and the shorter PHQ-2 are the most widely used versions in clinical practice.

Professor Robert Spitzer developed the PHQ-9 in the 1990 s based on the diagnostic criteria for MDD from the fourth edition of the DSM-IV. It is a depression screening tool containing nine items, scored according to the frequency of depressive symptoms experienced over the past two weeks. Each item is rated on a scale from 0 to 3, with the total score ranging from 0 to 27 [56]. The PHQ-9 quantifies symptom frequency and severity, assessing depression and providing valuable information for diagnosis and treatment decisions.

The PHQ-9 has been widely used in international studies to assess adolescent depressive symptoms, yielding consistent results across different cultural contexts [58]. In a randomized controlled trial involving high school students in Pennsylvania, the PHQ-9 demonstrated a sensitivity of 89% and specificity of 73%, indicating strong screening validity. Additionally, the PHQ-9 showed short-term test-retest reliability of 0.84, further confirming its stability in adolescent populations [59]. Additionally, a study conducted in Henan, China, with 471 school-aged adolescents (ages 14–18) found that the PHQ-9 had a Cronbach’s α of 0.85 and a test-retest reliability of 0.88, indicating good internal consistency and stability. Furthermore, the study identified a cutoff score 10 for optimal screening, with a sensitivity of 93.33% and specificity of 96.83%. The study also analyzed the gender influence on depression detection rates, revealing no significant gender differences. This phenomenon may be attributed to unique cultural factors in China, such as family dynamics, social support systems, and gender role expectations, which may influence emotional expression [60].

The Patient Health Questionnaire 2-item Depression Screen (PHQ-2) is a simplified version of the PHQ-9, consisting of the first two items of the PHQ-9 (core depressive symptoms) [61]. While it is brief and time-efficient, it cannot assess suicidal ideation and is more suitable for initial screening to identify individuals who may require further evaluation [62]. However, for high-risk populations, additional assessments with more detailed scales should be conducted [63]. A study involving 2364 adolescents aged 12 to 21 compared the PHQ-2 and PHQ-9 for screening. It found that the probability of a positive screen with the PHQ-9 was 1.4 times higher than with the PHQ-2 [64].

### Depression screener for teenagers (DesTeen)

The Depression Screener for Teenagers (DesTeen), developed by Gerd Schulte-Körne at the University of Munich, serves as a valid self-report screening tool for adolescents aged 13 to 16. It was created by reviewing existing adolescent depression questionnaires. Items were clustered based on six characteristic symptom groups of juvenile depression, including depressed mood, loss of interest, loss of energy, feelings of worthlessness, feelings of guilt, and cognitive symptoms. The physical symptoms were omitted, and suicide attempts as well as psychomotor symptoms were excluded due to their rarity in primary care. The DesTeen comprises 14 items organized into 6 dimensions, with each item offering four possible response options. Each item is scored on a scale of 0 to 3, reflecting the severity of symptoms, where higher scores indicate greater levels of depression. A cutoff score of 18 is used to identify depressive symptoms [65]. Respondents were asked to choose the option that best suited their situation based on their status in the past two weeks and completing the entire scale takes 5–10 min [66].

The DesTeen scale exhibits good psychometric properties, with high internal consistency (Cronbach’s α = 0.87) and a specificity of 0.80, demonstrating certain accuracy in identifying the target population [66]. A study conducted an assessment using DesTeen on 88 German adolescents, revealing that each item of DesTeen exhibited good discriminative ability and high internal consistency (Cronbach’s α = 0.91). Significant differences in total scores were observed between depressed and non-depressed adolescents, demonstrating the effectiveness of DesTeen in screening for adolescent depression within mental health care settings. Nevertheless, the sample was confined to local Germans and was of modest size, necessitating further validation to establish the generalizability of the findings [65].

A brief screening instrument is required to effectively implement screening in a pediatric setting. To this end, the original DesTeen was refined into an abbreviated five-item version (DesTeen-a), which includes items, each with an AUC value exceeding 0.75. Research findings indicate that this 5-item scale maintains the sensitivity of the original while demonstrating superior specificity, along with high overall accuracy and sensitivity [65, 66].

### Scale information overview

#### Summary of scale information

Eight adolescent depression scales were finally included in this study for the narrative review, and the essential information is shown in Table 1.

**Table 1:** Basics of original adolescent depression scales

Year of development	Scale abbreviation	Full name	Primary developers (Team)	Country of development	Age group
1960	HAMD	Hamilton Depression Rating Scale	Max Hamilton	United Kingdom	Not specified
1961	BDI	Beck’s Depression Inventory	A.T. Beck	United States	≥ 13
1977	CES-D	Center for Epidemiologic Studies Depression Scale	Radloff, L.S.	United States	≥ 14
1981	RADS	Reynolds Adolescent Depression Scales	William M. Reynolds	United States	12–18
1985	CDI	Children’s Depression Inventory	M. Kovacs	United States	7–17
2001	KADS	Kutcher Adolescent Depression Scale	Brooks, S.J. Kutcher, S.	Canada	12–17
2001	PHQ	Patient Health Questionnaire	Spitzer, R.L. Kroenke, K.	United States	Not specified
2011	DesTeen	Depression Screener for Teenagers	Gerd Schulte-Körne	Germany	13–16

The development of the 8 scales spans over five decades (1960–2010 s), primarily emerging from Western academic contexts, particularly the United States. This historical trajectory reflects the evolution from adult-focused assessments to specialized tools for adolescents.

#### The three phases of the historical development of adolescent depression scales

- The initial phase (1960s) saw foundational adult-oriented scales like the HAMD and BDI, which established quantitative assessment frameworks through clinical observation and cognitive theory. While not designed for youth, these instruments laid the groundwork for subsequent adolescent-specific adaptations.
- The rapid development phase (1980s) marked a critical shift toward adolescent mental health needs. CES-D emerged as an efficient epidemiological screening tool, while the RADS became the first developmentally sensitive instrument addressing emotional fluctuations and social behaviors in teenagers. Building on the BDI, the CDI adapted the BDI framework with simplified language to accommodate children’s cognitive levels, addressing a crucial gap in youth mental health assessment.
- Recent decades have focused on optimizing practicality and cross-cultural applicability. The PHQ was developed to meet the high demands for efficient screening in clinical and community settings, enabling rapid clinical triage. Additionally, the KADS was explicitly designed for the rapid screening of adolescent depression, integrating DSM diagnostic criteria and adolescent behavioral characteristics. Similar to KADS, the DesTeen is also specifically designed for adolescents and emphasizes the cognitive and emotional symptoms of adolescent depression.

#### Development background and theoretical basis of depression scales

The development of depression assessment tools reflects diverse theoretical foundations and practical needs.

The HAMD was originally developed for use in drug trials and emphasizes clinical observation and assessment. The BDI is anchored in Beck’s cognitive triad theory, which emphasizes negative self-evaluation, pessimistic future expectations, and negative cognition about the world. Meanwhile, the CES-D, designed for epidemiological research, adopts a symptom dimension model, making it particularly suited for measuring depression prevalence in general populations. The RADS focuses on adolescents’ emotional fluctuations and social behaviors, integrating adolescent psychological development theories. Building on the BDI’s framework, he CDI evaluates cognitive and behavioral characteristics, simplified language and added behavioral indicators. The KADS combines DSM-IV criteria with features of adolescent psychological development to optimize school-based screenings, The PHQ, grounded in DSM-IV diagnostic criteria, was developed as a simplified version of the PRIME-MD diagnostic tool. By utilizing a self-report format, it enhances screening efficiency, enabling rapid triage in primary care. The DesTeen concentrates on the cognitive and affective symptoms of adolescent depression, aligning with the diagnostic criteria of DSM-IV-TR. Its aim is to accurately identify and differentiate depression from other mental disorders, making it applicable in mental health care settings.

These scales have evolved from traditional clinical observation to integrating modern cognitive theories and diagnostic criteria, balancing clinical rigor with practical feasibility across settings.

#### Dimensional analysis of depression scales

The 8 adolescent depression scales included in this study exhibit varying dimensions, ranging from 2 to 6, with most tools adopting 4 dimensions. The specific details are shown in Table 2. In the early stages of scale development, scale development prioritized comprehensive symptom coverage through longer item lists. However, as the understanding of depression deepened, redundant items were optimized to improve the efficiency and accuracy of the scales. Too many items may increase the burden of assessment, while overly simplified scales may fail to capture the complexity of depression. Therefore, developers need to balance comprehensiveness and efficiency to ensure the applicability of the scale in various contexts.

This study categorizes the dimensions of the 8 scales into three main areas: physiological, psychological, and social. All scales evaluate psychological symptoms, while 6 include physical indicators like sleep disturbances or appetite changes, and 3 cover the social dimension. Only the BDI and CES-D stand out as the most holistic instruments, systematically addressing all three domains. The other scales focus more on specific domains, reflecting the pursuit of a balance between comprehensiveness and efficiency for different application contexts.

**Table 2:** Dimensional analysis of 8 common depression scales

Scale abbreviation	Dimensions	Items	Reliability (Cronbach’s alpha), specificity
HAMD	4 factors: Retarded depression, Agitated depression, Anxiety reaction, Somatic symptoms and insomnia	17(24)	–
BDI	4 dimensions: Depressive mood, Low self-evaluation, Somatic complaints, Social withdrawal	21(13)	0.91,0.82
CES-D	4 dimensions: Depressive mood, Positive mood, Somatization, Interpersonal problems	20(13/10)	0.88–0.89,–
RADS	4 dimensions: Negative mood, Anhedonia/Negative emotions, Negative self-evaluation, Somatic complaints	30	0.92–0.96,–
CDI	5 dimensions: Low self-esteem, Negative emotions, Anhedonia, Inefficiency, Interpersonal problems	27(10)	0.86,0.84
KADS	KADS-11 has 2 factors: Lack of motivation, Restlessness	16(11/6)	0.84,0.71
PHQ	Single dimension, 3 common factors: Self-evaluation factor, Emotional factor, Somatization factor	9(2)	0.85,0.73
DesTeen	6 dimensions: depressed mood, loss of interest, loss of energy, feelings of worthlessness, feelings of guilt and cognitive symptoms	14(5)	0.87,0.8

## Discussion

Adolescent depression presents unique challenges due to changes in physiological development and cognitive growth, which lead to the diversity and variability of symptoms and emotional expressions. Given that adolescents are often perceived as impulsive, overly self-focused, and particularly susceptible to social pressures [67], they may exhibit depression-like symptoms when confronted with academic pressures or experiencing normal pubertal emotional fluctuations. This propensity, coupled with the transient nature of adolescent emotions, often leads to a higher false-positive rate in screening results [68]. Furthermore, positive screening outcomes may engender stigmatization [69], potentially adversely affecting both the mental well-being and social interactions of adolescents. Thus, selecting context-appropriate tools is critical. This narrative review analyzes 8 commonly used adolescent depression scales through reliability and validity assessments, age suitability, and symptom coverage, offering evidence-based guidance for clinical practice.

Firstly, the HAMD considered the “gold standard” for assessing the severity of depression, has high reliability and validity in adults. However, given the immature physical and psychological development of adolescents, there are significant differences in how depression manifests, how adolescents perceive the severity of depressive symptoms, and how assessment and intervention approaches are used compared to adults. Williams et al. pointed out the scale’s limitations in terms of its dimensionality [11], though its validation in multiple languages and cultures suggests potential applicability in adolescent populations. Consequently, the application and cross-cultural validation of relevant methods and tools for adolescents require further in-depth research.

The BDI and CES-D scales provide the most comprehensive coverage, encompassing physiological, psychological, and social dimensions. The BDI focuses on emotional experiences and physiological responses, addressing aspects such as mood, sleep, appetite, and other physical changes. Initially designed for adults, research by Klein et al. highlighted that some abstract items may pose challenges for adolescents [70], particularly younger students and those with limited cognitive abilities. These individuals often struggle to understand abstract items that involve complex emotional introspection and metaphorical expressions, which in turn affects the accuracy of the assessment. Additionally, adolescents face a social environment that is significantly different from that of adults, encountering unique stressors such as academic pressure, peer relationships, and physical changes during puberty. Consequently, the original item settings of the BDI may not fully capture the depressive manifestations triggered by these adolescent-specific stressors, potentially compromising the scale’s accuracy in assessing adolescent depression. In contrast, the CES-D scale is particularly suited for large-scale epidemiological studies. Its balanced approach to emotional and behavioral symptoms makes it practical for population assessments, but it also faces challenges in distinguishing between emotional and somatic symptoms. Research by Yang Wenhui et al. highlighted the risk of false positives, particularly for atypical symptoms. These can be effectively assessed using the Inventory of Depressive Symptomatology (IDS), a tool that emphasizes the detection of atypical symptoms through detailed ratings of the primary symptoms of depression [71]. Therefore, while the BDI is more suitable for clinical use, the CES-D is better for broad screening.

The RADS was explicitly designed for adolescents, with a comprehensive set of items and strong reliability and validity, particularly in emotional screening. However, the large number of items could lead to fatigue effects among respondents, especially when dealing with severely depressed individuals who have limited energy and attention. This fatigue not only affects their patience and concentration when answering questions but also may result in neglecting or randomly responding to items related to subsequent behaviors and somatic symptoms, thereby complicating the recognition of these symptoms. Steven Regeser Lopez also indicated that existing scales have limitations in cross-cultural settings [72], as they may not accurately capture the actual psychological states of patients in different cultures.

The CDI, a classic tool for screening depression in children and adolescents, covers a multidimensional assessment of depressive symptoms and is suitable for individuals aged six and above. However, given the varying cognitive development levels across age groups, its applicability has been debated, especially in detecting subtle emotional fluctuations. Stockings et al. noted that its five-factor structure includes externalizing behaviors and anxiety [8], which introduces some uncertainty regarding its specificity to depression. Specifically, for preadolescents aged 6 to 12 years, the relatively abstract item “I feel like crying” may be prone to misinterpretation, as preadolescents often have a limited emotional vocabulary at this stage. This limitation can lead to false negative results during the screening process.

The KADS, specifically designed for adolescents, is widely used due to its simplicity and focus on the unique psychological characteristics of this age group, such as self-esteem and social relationships. Its straightforward language makes it suitable for adolescents across various age groups. However, while the KADS is efficient and concise, it may fall short compared to the BDI in capturing the complexity of adolescent depressive symptoms, especially atypical ones such as emotional fluctuations. A study by Amin found that while the scale generally demonstrates good reliability and validity, some items may fail to adequately assess depression symptoms in specific gender groups, resulting in scoring discrepancies [73]. This indicates that the Kutcher scale’s applicability across different populations requires further investigation and improvement. Furthermore, KADS has limited cross-cultural validation, which may impact its applicability in diverse cultural contexts.

The PHQ is widely used for rapid screening and monitoring treatment effectiveness, especially in time-sensitive environments. It assesses depressive symptoms with nine items, offering high operational simplicity. However, its design emphasizes somatic symptoms and essential emotional evaluation, making it difficult to fully capture the complex emotional changes seen in adolescents, such as irritability and emotional fluctuations. As a result, adolescent who do not exhibit typical somatic symptoms of depression but experience significant emotional issues may be overlooked during the screening process. Sekhar et al. pointed out that while the PHQ facilitates widespread screening in schools, relying solely on the scale may miss students without typical depressive symptoms [59]. Considering the individual’s life circumstances and social background, a more comprehensive assessment is needed.

The DesTeen, a screening tool specifically developed for adolescent depression, demonstrates robust validity in mental health settings and significantly improves diagnostic accuracy. It consists of 14 items that focus on cognitive and affective symptoms while omitting somatic aspects, presenting a clear and comprehensible structure. A concise 5-item version, DesTeen-a, is also available for rapid assessments. However, Antje-Kathrin Allgaier and colleagues have raised concerns about its generalizability due to a limited, exclusively German-speaking sample [65]. Furthermore, the independent efficacy of DesTeen-a and its capacity to track severity fluctuations throughout treatment remain to be clarified.

Rapid and efficient screening tools are crucial in a school setting, where the number of students is large and time and resources are limited. Compared to the other seven scales, the CES-D has concise and clear items, efficiently reflecting recent changes in students’ emotional states, making it suitable for large-scale depression screening in schools. It can quickly identify students who may be at risk of depression, but caution is needed regarding false positive results. For younger students, the CDI is more appropriate, as is specifically designed for children and adolescents, using simple language that is easier for them to understand and requiring a short completion time. It can assess depressive symptoms multidimensionally, aiding teachers and school psychologists in gaining a preliminary understanding of students’ emotional states. When choosing between the two tools, it is essential to consider factors such as the age distribution of students, available psychological counseling resources, and follow-up capacity. If a large proportion of lower-grade students is present and the psychological counseling staff is limited, a combination of initial screening with the CDI and follow-up interviews by available staff might be a way to balance cost and effectiveness. Conversely, if the goal is to conduct large-scale, rapid screening followed by a detailed identification process, the CES-D can be employed to preliminarily identify at-risk groups.

In primary care settings, balancing rapid assessment with accurate diagnosis is key. The PHQ-9 is preferred due to its brief completion time and focus on symptom assessment relevant to clinical treatment decisions. This helps doctors determine whether further intervention or referral is necessary. While its content is clear and accessible, the PHQ-9 may not fully capture the complex emotional changes in adolescents, as it was initially designed for adults. For doctors seeking a more comprehensive understanding of a patient’s depression and wishing to develop personalized treatment plans, the BDI is a better tool. It provides an in-depth assessment of mood, sleep, and appetite changes, quantifying the severity of depressive symptoms, which aids in formulating personalized treatment plans. It is suitable for patients with more complex symptoms or those requiring more precise treatment plans, although its more detailed assessment content may require a certain level of understanding and expressive ability from patients. Doctors can combine both tools based on actual situations to more comprehensively evaluate a patient’s condition.

In professional mental health services, accuracy and comprehensiveness are paramount. The RADS, designed specifically for adolescents, is comprehensive and in-depth, with higher reliability and validity compared to other scales. Despite potential respondent fatigue, its advantages are significant. The HAMD, regarded as the “gold standard” for assessing the severity of adult depression, still holds authority in judging the severity of adolescent depression. However, compared to the RADS, it lacks rigorous research and practical testing specifically for the adolescent population, necessitating further research on its application and cross-cultural validation. For professionals with rich experience in adult depression assessment, the HAMD can be a secondary choice when first encountering adolescent depression cases. The RADS can be effectively utilized as the principal instrument to conduct a comprehensive exploration of the emotional issues prevalent among adolescents. Meanwhile, the HAMD can be deployed to corroborate and refine the assessment of the severity of pivotal symptoms, thereby enhancing the overall diagnostic precision.

When selecting depression assessment tools, clinicians and researchers should prioritize three dimensions: assessment purpose, population characteristics, resource constraints. For large-scale school screenings with time limitations, brief self-report tools like the CES-D (ages ≥ 14) or CDI (ages 7–17) are optimal for initial risk identification. If resources allow, a two-stage approach combining CDI screening with clinician interviews improves accuracy. In primary care, the PHQ-9 serves as a pragmatic first-line tool for triaging adolescents, while the BDI-II is preferable when symptom complexity demands granularity. For professional mental health settings, the RADS should be the cornerstone for adolescent assessments due to its developmental sensitivity; the HAMD may supplement severity evaluation but requires cautious interpretation given its adult-centric validation.

The development of existing depression scales largely originates from Western cultural contexts, and their theoretical frameworks—such as the DSM-5’s core criterion of “depressed mood”—may fail at capturing the symptom expression patterns of non-Western populations. In East Asian cultures, adolescents with depression tend to express psychological distress through somatic symptoms. Research indicates that when assessed using the CES-D and BDI scales, Chinese and Japanese individuals exhibit significantly higher mean scores on somatic symptoms compared to Americans [74–76]. This discrepancy may stem from the emphasis on collectivism in East Asian cultures, which discourages the overt expression of strong emotions, particularly negative ones, leading individuals to indirectly express mental health needs through somatic complaints [76, 77]. Due to this culturally specific expression, which may lack core depressive symptoms defined in Western standards, such as depressed mood and anhedonia, the results of depression scale assessments may be subject to bias. Similarly, in South Asian cultures, depressive emotions are often regarded as factors that individuals can control, and they are closely associated with stigma. This stigmatization of psychological symptoms may lead adolescents to deliberately conceal their symptoms during scale assessments [78], resulting in false negatives and the accuracy of diagnosis. In Russian culture, the expression of negative emotions is considered normal and widespread, non-specifically associated with depression. This cultural difference may lead to an increased response threshold of subjects for scale items, potentially underestimating their association with depression [79], which could impact the applicability and accuracy of these scales among Russian populations.

Moreover, cultural factors such as religion significantly influence suicidal thoughts and behaviors. For instance, in Catholic cultures, suicide is regarded as a sin against religious doctrine, a belief that may introduce bias in the assessment of related items [80]. Therefore, when using Western scales to diagnose adolescents from non-Western cultural backgrounds, misdiagnosis may occur if cross-cultural factors are not considered. It is noteworthy that even culturally adapted depression scales exhibit significant differences in performance. For example, the CES-D scale has shown excellent performance among American Indian adolescents and South Africans in terms of reliability, validity, and clinical utility [81, 82]; while the PHQ, when applied to Chilean and Australian populations, demonstrates good internal consistency, sensitivity, negative predictive value, and structural validity, but performs poorly in terms of specificity and positive predictive value, leading to false-positives [83, 84]. This underscores the need for critical evaluation during the cross-cultural application, emphasizing the design and development of culture-specific items and adjustments to scoring algorithms to ensure diagnostic accuracy and effectiveness.

Future research should address three key challenges in adolescent depression assessment. First, we need to improve cultural adaptability of existing measurement tools. While current scales have shown progress, they require better translation methods and cultural adjustments to ensure consistent results across different populations. Advanced statistical techniques like multi-group factor analysis can help verify whether these tools work equally well in diverse cultural settings [85]. Second, assessment tools must account for overlapping symptoms with common co-occurring conditions. For instance, the hyperactivity symptoms of ADHD(Attention-deficit hyperactivity disorder) may mask the social withdrawal characteristic of depression, while the abnormal eating behaviors associated in eating disorders can interfere with accurate evaluation. To address this, future scales should incorporate items with disease specificity for differential diagnosis. Additionally, considering the unique psychological developmental characteristics of adolescents, it is essential to add assessment items for emotional fluctuations, social withdrawal, and atypical symptoms. Finally, we need to modernize assessment methods. Traditional paper surveys are becoming outdated with today’s youth. Future efforts could focus on developing digital and gamified versions of these scales, leveraging technology to increase adolescent engagement while reducing self-report biases.

### Limitations

First, the majority of current studies predominantly use clinical and school samples. However, these samples may not fully represent the heterogeneous adolescent population, thereby engendering potential risks of sampling bias. Second, our review’s predominant focus on English and Chinese literature introduces potential language and geocultural biases. Scale validation studies in other linguistic or regional contexts may have been overlooked, limiting the generalizability of findings to non-Western populations. Third, most of the existing scales for adolescent depression research primarily adopt the cross-sectional study method, which makes it challenging to capture the dynamic changes of adolescent depression. Methodologically, our inclusion criteria prioritized widely cited scales, potentially omitting emerging tools with limited validation data. Lastly, while we highlighted cross-cultural challenges, the review did not systematically evaluate measurement invariance across populations—a critical gap for future research.

## Conclusions

The findings of this narrative review indicate that each of the 8 depression scales has its applicable scenarios and limitations. In clinical practice, the selection of a scale should not only consider its reliability and validity but also consider whether the item design is suitable for the cognitive level of adolescents, the time required to complete the scale, and the specific needs of the assessment context. Additionally, this study highlights the issues related to the applicability of these scales across different cultural backgrounds.

## Data Availability

No datasets were generated or analysed during the current study.

## Abbreviations

ADHD: Attention-deficit hyperactivity disorder
BDI: Beck Depression Inventory
CDI: Children's Depression Inventory
CES-D: Center for Epidemiologic Studies Depression Scale
DesTeen: Depression Screener for Teenagers
DSM-IV: The fourth edition of the Diagnostic and Statistical Manual of Mental Disorders
EFA: Exploratory Factor Analysis
HAMD: Hamilton Depression Scale
IDS: Inventory of Depressive Symptomatology
KADS: Kutcher Adolescent Depression Scale
MDD: Major depressive disorder
MDE: Major Depressive Episode
MGCFA: Multi-Group Confirmatory Factor Analysis
MINI KID: Mini International Neuropsychiatric Interview for Children and Adolescents
MMPI: Minnesota Multiphasic Personality Inventory
PHQ: Patient Health Questionnaire
PPV: Positive predictive value
PRIME-MD: Primary Care Evaluation of Mental Disorders
RADS: Reynolds Adolescent Depression Scales
RDC: Research Diagnostic Criteria
SCAS: Spence Children's Anxiety Scale
SDS: Self-Rating Depression Scale
